# Developmental and adult characterization of secretagogin expressing amacrine cells in zebrafish retina

**DOI:** 10.1371/journal.pone.0185107

**Published:** 2017-09-26

**Authors:** Stefanie Dudczig, Peter David Currie, Patricia Regina Jusuf

**Affiliations:** 1 Australian Regenerative Medicine Institute, Monash University, Clayton, VIC, Australia; 2 School of Biosciences, University of Melbourne, Parkville, VIC, Australia; National Eye Centre, UNITED STATES

## Abstract

Calcium binding proteins show stereotypical expression patterns within diverse neuron types across the central nervous system. Here, we provide a characterization of developmental and adult secretagogin-immunolabelled neurons in the zebrafish retina with an emphasis on co-expression of multiple calcium binding proteins. Secretagogin is a recently identified and cloned member of the F-hand family of calcium binding proteins, which labels distinct neuron populations in the retinas of mammalian vertebrates. Both the adult distribution of secretagogin labeled retinal neurons as well as the developmental expression indicative of the stage of neurogenesis during which this calcium binding protein is expressed was quantified. Secretagogin expression was confined to an amacrine interneuron population in the inner nuclear layer, with monostratified neurites in the center of the inner plexiform layer and a relatively regular soma distribution (regularity index > 2.5 across central–peripheral areas). However, only a subpopulation (~60%) co-labeled with gamma-aminobutyric acid as their neurotransmitter, suggesting that possibly two amacrine subtypes are secretagogin immunoreactive. Quantitative co-labeling analysis with other known amacrine subtype markers including the three main calcium binding proteins parvalbumin, calbindin and calretinin identifies secretagogin immunoreactive neurons as a distinct neuron population. The highest density of secretagogin cells of ~1800 cells / mm^2^ remained relatively evenly along the horizontal meridian, whilst the density dropped of to 125 cells / mm^2^ towards the dorsal and ventral periphery. Thus, secretagogin represents a new amacrine label within the zebrafish retina. The developmental expression suggests a possible role in late stage differentiation. This characterization forms the basis of functional studies assessing how the expression of distinct calcium binding proteins might be regulated to compensate for the loss of one of the others.

## Introduction

Calcium is a signaling molecule involved in many cellular processes. In neurons of the central nervous system (CNS) calcium contributes to growth and differentiation including synaptogenesis, cell death, neurite arbor size and complexity, extracellular guidance and normal neural functioning by controlling neurotransmitter release and cell membrane excitability [1–5]. Calcium imbalance can contribute to neurodegenerative diseases such as Alzheimer’s [6–10]. Due to the importance of calcium in regulating such diverse neural functions, calcium homeostasis is highly regulated in part through a variety of different widely expressed calcium binding proteins (CaBP) [11]. These are broadly subdivided into calcium buffers or sensors, defined by their molecular and signaling properties, though individual proteins can have functions spanning both categories [4, 12]. Some individual proteins can also act as both buffer and sensor depending on the calcium concentration [13, 14]. Passive calcium buffers such as calbindin, calretinin, and parvalbumin bind calcium with high affinity to maintain intracellular concentration of free calcium [15]. These are generally expressed in phylogenetically preserved tissues within distinct neuron subtypes in rodent, primate and human [16–18]. In contrast, calcium sensors such as calmodulin bind calcium with lower affinity to cause a conformational change and influence downstream signaling pathways (Burgoyne and Haynes 2012?). Such calcium sensors show more widespread expression across neuron types [13]. The many different identified CaBPs have different roles and substrate specificity [6–10].

Secretagogin is a member of the six EF-hand CaBP and shows high homology to calcium buffers including calretinin and calbindin [19]. However, secretagogin binds four Ca2+ with low affinity and subsequently undergoes conformational changes to modulate exocytosis signaling via binding to the synaptosomal associated protein 25 (SNAP25), similar to a calcium sensor [4, 20–22], though recent biochemical data suggests that it could act as a calcium buffer in oxidizing envrionemnt such as in the ER [23]. Initially cloned from pancreatic islets of Langerhans and neuroendocrine cells [19], secretagogin has meanwhile been found expressed in a wide variety of CNS neurons found in the olfactory bulb, hippocampus, telencephalon, cerebellum, hypothalamus, neocortex and the retina of different vertebrates [19, 22, 24–32]. The neuronal subtype identity of secretagogin varies between vertebrates even within the rodent or primate orders [33]. Secretagogin mostly labeling distinct subpopulations of neurons though it shows occasional co-localization with other CaBPs such as calbindin, calretinin or parvalbumin, with extensive co-localisation reported in few CNS regions [25, 26, 33–37]. The expression level of secretagogin is dynamic and can for instance be regulated by glucose [25, 38]. While its levels are not altered in Alzheimer’s brains [39], the density of a subpopulation of SCGN expressing neurons is decreased in Alzheimer’s disease [28]. SCGN directly interacts with Tau in a Ca^2+^ dependent manner and shows neuroprotective properties [27, 40]. SCGN has also been implicated in modulating the stress response, as it influences cortocotropoin releasing hormone and corticosterone levels [41]. Additionally, SCGN is upregulated within the rostral migratory stream where it aids neuroblast migration by controlling externalisation of matrix metalloprotease-2 [42]. Thus, while the neuronal subtype expression (and subcellular expression) of secretagogin and comparison with other identified calcium binding proteins is continuing to be established, the function within distinct CNS regions is only starting to be identified, and the functional significance of co-expression of multiple calcium binding proteins remains unclear (reviewed by [22]).

As one of the best characterized and highly organized part of the CNS, the neural retina in the eye is an important model system for understanding development, organization and regeneration of CNS neurons and assessing protein functionality in neurons and neural circuits. In the vertebrate retina, the neuronal expression of CaBPs varies between species [43–47]. Similarly, secretagogin expression has been identified in distinct subtypes of bipolar and amacrine cells, with species-specific differences [30, 31, 34, 48–50]. In the zebrafish vertebrate retina, the Calcium Binding Proteins CaBP1a, 1b, 2a, 4b, 5b, Caln1 and 2 were recently shown to be developmentally expressed in distinct neural subpopulations primarily after differentiation of the central retina, suggesting a role in mature fully developed neurons [11]. As the zebrafish retina is an established developmental model for studying central nervous system generation and is particularly amenable to linking protein expression, their functional role and dynamic neurodevelopmental processes, the goal of the present study was to describe the developmental expression pattern of SCGN and CaBP co-expression pattern within the retina of zebrafish. Combined with the ease of functional gene manipulations, this work can aid with greater insight into the role of these proteins and their regulation during neurogenesis.

## Materials and methods

### Animals

Zebrafish were maintained and bred at 26.5°C, and embryos of either gender used for experiments were raised at 28.5°C and staged as previously described [51] in hours postfertilization. This study was carried out in accordance with the provisions of the Australian National Health and Medical Research Council code of practice for the care and use of animals. The protocol was approved by the local ethics committees at Monash University and the University of Melbourne.

### Immunohistochemistry

Zebrafish embryos (1 or 2 dpf) and larvae (3, 4 or 5 dpf) were fixed in 4% paraformaldehyde in phosphate buffered saline pH 7.4 at room temperature for 3 hours or overnight at 4°C. Zebrafish were rinsed with phosphate buffered saline, cryoprotected in 30% sucrose, embedded in OCT (Tissuetek) and cryosectioned at 14 μm thickness. All immunohistochemistry steps were performed at room temperature.

Sections were incubated in primary antibodies diluted in 0.2% triton X-100 in phosphate buffered saline overnight (2 nights for secretagogin antibody). Sections were rinsed in PBS and incubated in secondary antibodies for 2 hours at room temperature. Secondary antibodies diluted in 0.2% triton X-100 in phosphate buffered saline were goat or donkey anti-mouse, anti-rabbit or anti-sheep IgG conjugated to Alexa 488 or 546 fluorophores (1:500 dilution, Molecular Probes). After further rinses, nuclei were counterstained with 4’,6-diamidino-2-phenylindole (DAPI) and sections were coverslipped in Mowiol.

### Antibody characterization

The rabbit anti-gamma-aminobutyric acid (GABA) antibody (RRID: AB_477652, 1:2,000) was generated against GABA conjugated to bovine serum albumin. It is used to label GABAergic amacrine cells. The antibody specifically recognizes GABA in a dot blot assay (manufacturer’s datasheet, species not mentioned). The structure of GABA is identical in all vertebrate species. Within the zebrafish retina, the staining pattern obtained using this GABA antibody is comparable with that obtained using antibodies against GAD65 and GAD67, the glutamic acid decarboxylates, which are the enzymes that convert glutamate to GABA [52].

The rabbit anti-calbindin (CB) D-28K antibody (RRID: AB_213554, 1:500) generated against purified bovine cerebellum calbindin D-28K protein recognizes the expected 28 kDa band in Western blots in zebrafish brain homogenate. Calbindin immunoreactivity in zebrafish is abolished when the primary antibody is preabsorbed with recombinant rat calbindin [53].

The rabbit anti-calretinin (CR) antibody (RRID: AB_2068506, 1:2,000) generated against recombinant rat calretinin specifically recognizes a 28kDa Western blot band in zebrafish brain homogenate [53].

The mouse anti-parvalbumin (PV) antibody (RRID: AB_2174013, 1:1,000) specifically stains Ca2+ bound forms of parvalbumin and recognizes a 12 kDa protein in Western blots of mouse brain lysate (manufacturer’s datasheet) and whole zebrafish homogenate (our data not shown).

The mouse monoclonal anti-sex determining region Y–box 2 (Sox2) antibody clone Sox2-6 (RRID: AB_10603254) (1:200) has been generated against amino acids 32–43 of human SOX2 and was used to label a subpopulation of amacrine cells.

The goat anti-choline acetyl transferase (ChAT) antibody (RRID: AB_2079751, 1:500) was made against human placental enzyme ChAT and recognizes a specific 70 kDa band on Western blots of mouse brain lysate (manufacturer’s datasheet). In zebrafish, immunoreactivity is abolished when the antibody is preabsorbed with recombinant rat ChAT [54].

The rabbit and sheep anti-secretagogin (SCGN) antibodies (RRID: AB_2034060 and RRID:AB_2034062, both 1:4,000) were generated against the full length recombinant human secreatgogin + 10 amino acid N-terminal histidine-tag. The antibodies show identical staining in zebrafish (data not shown). In mammalian species both of these antibodies label the same cells as a non-commercial secretagogin antibody that recognize the 32 kDa predicted band in mouse retina and cerebellum [30, 31].

The mouse anti-tyrosine hydroxylase (TH) antibody (RRID: AB_2201528, 1:1,000) generated against TH purified from PC12 cells and labels dopaminergic neurons. The antibody is raised against a rate-limiting enzyme during dopamine production. This commercial antibody was raised against TH purified from rat PC12 cells. It recognizes an epitope outside of the regulatory N-terminus, and a 62 kDa band in immunoblots of zebrafish brain [53]. In zebrafish CNS this antibody exclusively labels TH1 as shown by colocalization with th1, but not th2 in situ hybridization [55]. Additionally, immunolabeling with this antibody is abolished following morpholino mediated knockdown specifically of Th1 in embryonic zebrafish CNS [56].

The rabbit anti-neuropeptide Y (NY) antibody (RRID: AB_572253, 1:500)) was generated against NY coupled to bovine thyroglobulin with glutaraldehyde and specifically stains NY in the rat central nervous system, which is blocked by preabsorption (manufacturer’s datasheet). NY is a small 36 amino acid neurotransmitter that is highly conserved across all vertebrates, with zebrafish NY showing 89% homology to the human NY (Soderberg et al., 2000). In goldfish, preabsorption of this antibody with rat NY abolishes immunoreactivity in the brain and pituitary as shown by immunoblotting, enzyme immunoassay and radioassay [57].

The rabbit anti-serotonin (5-HT) antibody (RRID: AB_477522) was generated against serotonin creatinine sulphate complex conjugated with formaldehyde to bovine serum albumin. It reacts with serotonin-containing fibers in rat brain and specific staining is inhibited by preabsorption with serotonin or serotonin-BSA (manufacturer’s datasheet). 5-HT is identical in all vertebrate species [54]. Preabsorption with 5HT-BSA conjugate completely abolishes zebrafish staining [53].

### Imaging and analysis

Processed retinal sections were photographed at the Z1 Axioscope using Axiovision software (Zeiss) using the Apotome, or at the Leica DM6000 using Metamorph software. Images were processed in Adobe Photoshop (brightness and contrast adjustments) and figure panels combined using Adobe Illustrator. Numbers of embryos / cells analyzed are indicated in the relevant results section.

## Results

### Developmental expression of secretagogin in retinal neurons

**Fig 1:**
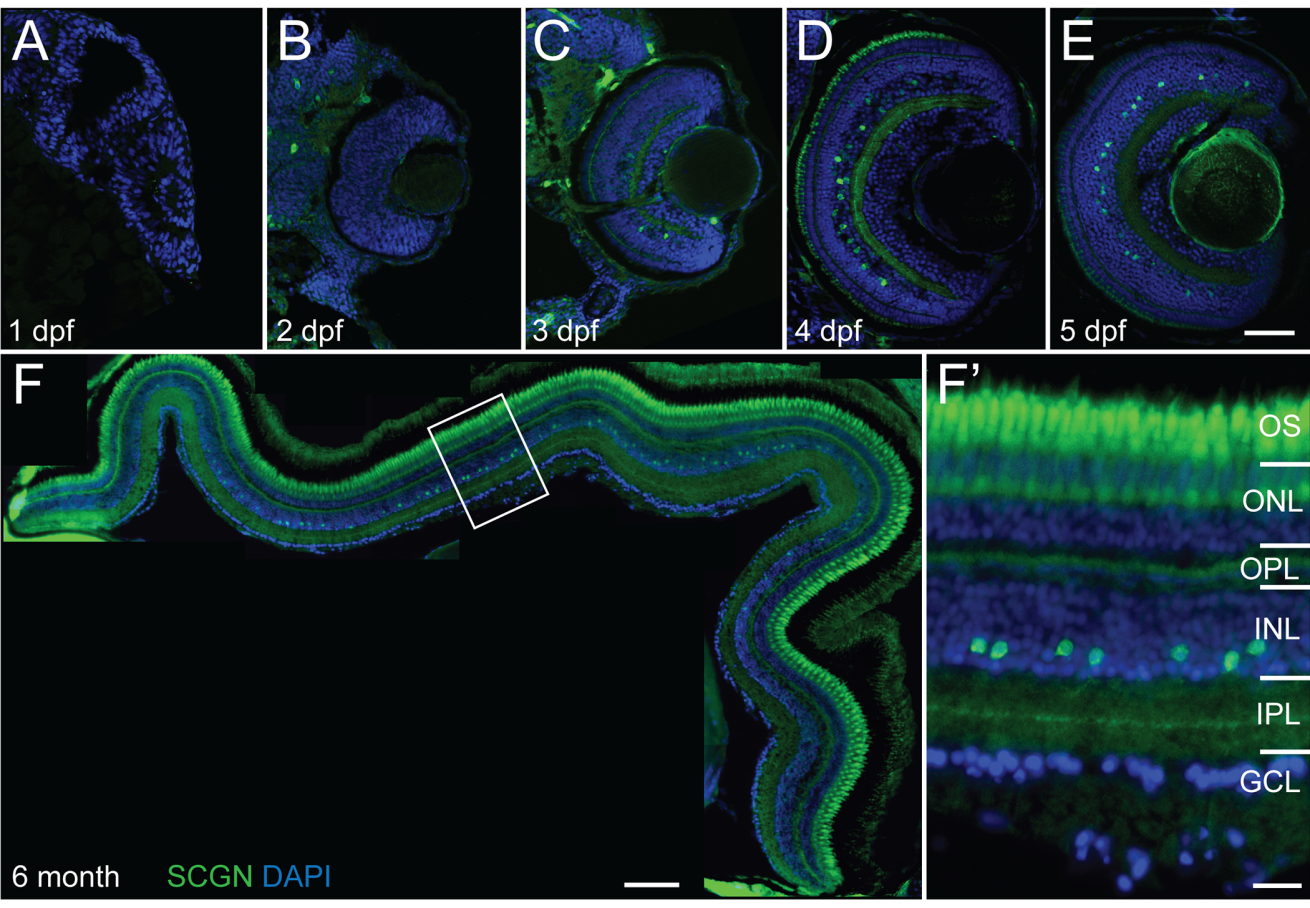
Secretagogin expression in embryonic and adult zebrafish retina. Micrographs of vertical sections through zebrafish retina immunohistochemically labeled for secretagogin (SCGN–green) with nuclei counterstained by DAPI (blue). (A–E) Sections through retinas at 1–5 days post fertilization (dpf) show earliest secretagogin positive cells detected at 3 dpf (C) and maintained at subsequent days. (F) Collage through retinal section in 6 month old zebrafish. Secretagogin expression in the amacrine layer in the inner half of the inner nuclear layer (INL) remains strong throughout adulthood. (F’) Higher magnification inset of boxed region in F shows secretagogin labeled with stained processes showing monostratified band in the center of the inner plexiform layer (IPL). OS: outer segments; ONL: outer nuclear layer; OPL: outer plexiform layer; GCL: ganglion cell layer. Scale bar (E) for A-E is 50 μm, scale bar (F) is 100 μm, scale bar (F’) is 20 μm.

The temporal expression of the calcium binding protein secretagogin was quantified to understand at which stage of neural development or differentiation such important Ca^2+^ regulators are starting to become functional. In retinal zebrafish sections labeled for secretagogin immunoreactivity, the first labeling could be observed in retinal neurons at 3 days postfertilization (dpf) (Fig 1C). There was no SCGN labeling at 1 dpf (Fig 1A), when the developing retina consists purely of dividing progenitor cells [58], just prior to the first neurons differentiating at 28 hpf, nor at 2 dpf (Fig 1B), when differentiated neurons are already present within the retina. The SCGN+ neurons were found in the inner half of the inner plexiform layer (INL), which contains the amacrine interneurons. Amacrine cells in this layer start differentiating already at 35 hours postfertilization [59] and by 3 dpf, the central region of the zebrafish consists completely of differentiated neuron types already synaptically connected to form appropriate neural circuits [58]. However, SCGN immunoreactivity first observed at 3 dpf, was still very sparse in only 1.8% ± 0.77% SEM (n = 291 cells from 8 retinas) of neurons in this layer. The proportion of SCGN+ cells significantly increased (p-value = 5 x 10^−6^, Student’s t-test) between 3 and 4 dpf (9.8% ± 0.9% SEM, n = 426 cells from 10 retinas) and then remained stable showing no significant change (p-value = 0.47, Student’s t-test) from 4 to 5 dpf (9.0% ± 0.76% SEM, n = 327 cells from 10 retinas) of neurons in this layer Fig 1D–1F). The timing and location of this developmental expression pattern suggests that secretagogin has little role in the neurogenesis of the retinal neurons, but carries out its function after neural cell differentiation and synaptic circuit formation.

### Secretagogin immunoreactive amacrine cells

**Fig 2:**
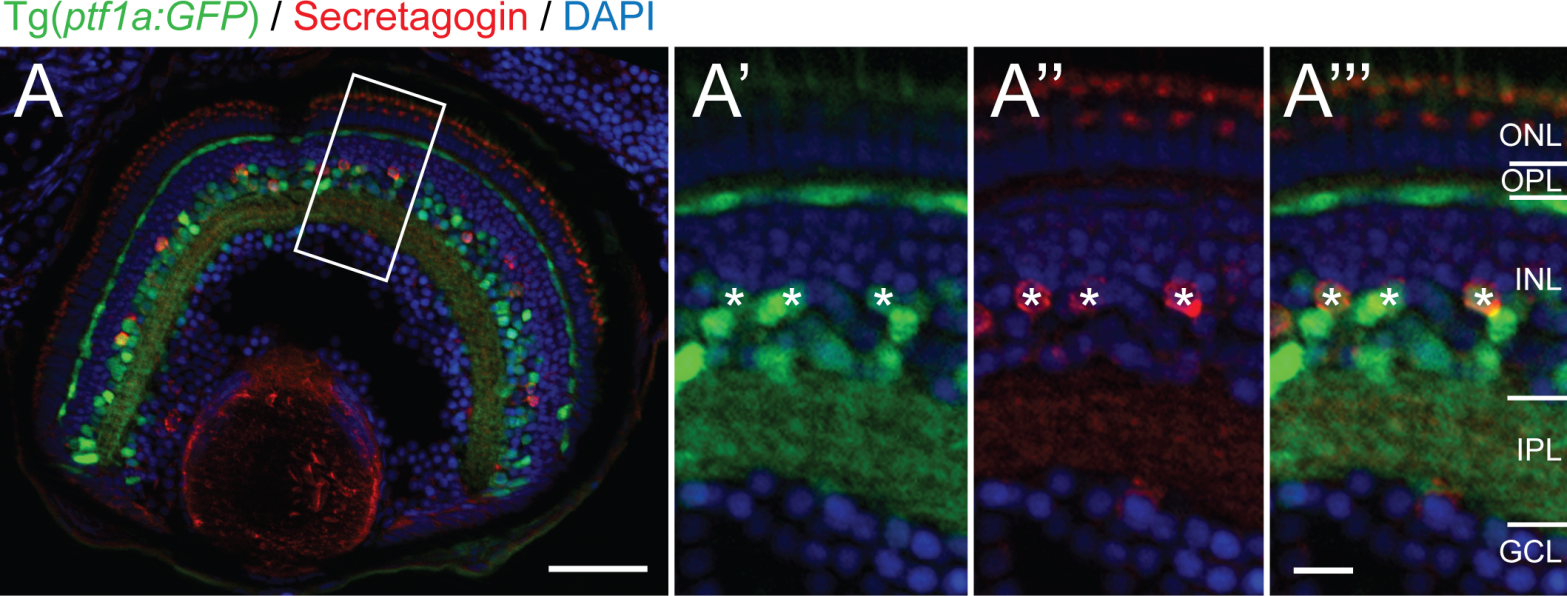
Secretagogin positive cells co-label with the Ptf1a:GFP amacrine marker in the inner nuclear layer. Micrograph at 5 days postfertilization showing secretagogin immunostained Tg(*ptf1a*:*GFP*) zebrafish retinas. Higher magnification of boxed view of boxed inset shows co-localization (asterisks) of SCGN+ (red) and Ptf1a:GFP+ (green) within individual cells marked in the green (A’), red (A”) and double (A”‘) channels.). ONL: outer nuclear layer; OPL: outer plexiform layer; INL: inner nuclear layer; IPL: inner plexiform layer; GCL: ganglion cell layer. Scale bar (A) is 50 μm, scale bar (A”‘) for A’–A”‘ is 10 μm.

Given that SCGN+ cells were confined to the inner half of the INL, the Tg(*ptf1a*:*GFP*) transgenic line was used to label amacrine cells usually located within this layer at 5 dpf. Quantification of co-localization confirmed that most SCGN+ cells also expressed Ptf1a:GFP transgene (86% ± 1.9% SEM, n = 93 cells from 7 larvae). The SCGN+ cells never co-localized with Ptf1a:GFP labeled horizontal cells in the outermost cellular layer of the INL and were never found in Ptf1a:GFP displaced amacrine cells in the ganglion cell layer (Fig 2).

Within the zebrafish retina the amacrine cells occupy the three innermost rows of nuclei in the inner nuclear layer. SCGN+ cells represent 5.2% ± 0.42% SEM of cells in this amacrine layer (n = 1140 cells from 15 larvae). The distribution of SCGN+ labeled amacrine cell somas was 7% in the innermost row closest to the inner plexiform layer, 20% in the middle row and 73% in the outermost row of the amacrine layer. Labeling of SCGN+ cell processes in the inner plexiform layer (IPL) become confined to a single band in the center of the layer at 54.3% ± 0.53% SEM IPL depth (n = 16 ROIs from 4 adult retinas) in adults (Fig 1F’). The location of the cell bodies and neurite stratification pattern of SCGN+ cells suggests that these comprise a population of monostratified amacrine interneurons.

### Distribution and density of secretagogin immunoreactive neurons

**Fig 3:**
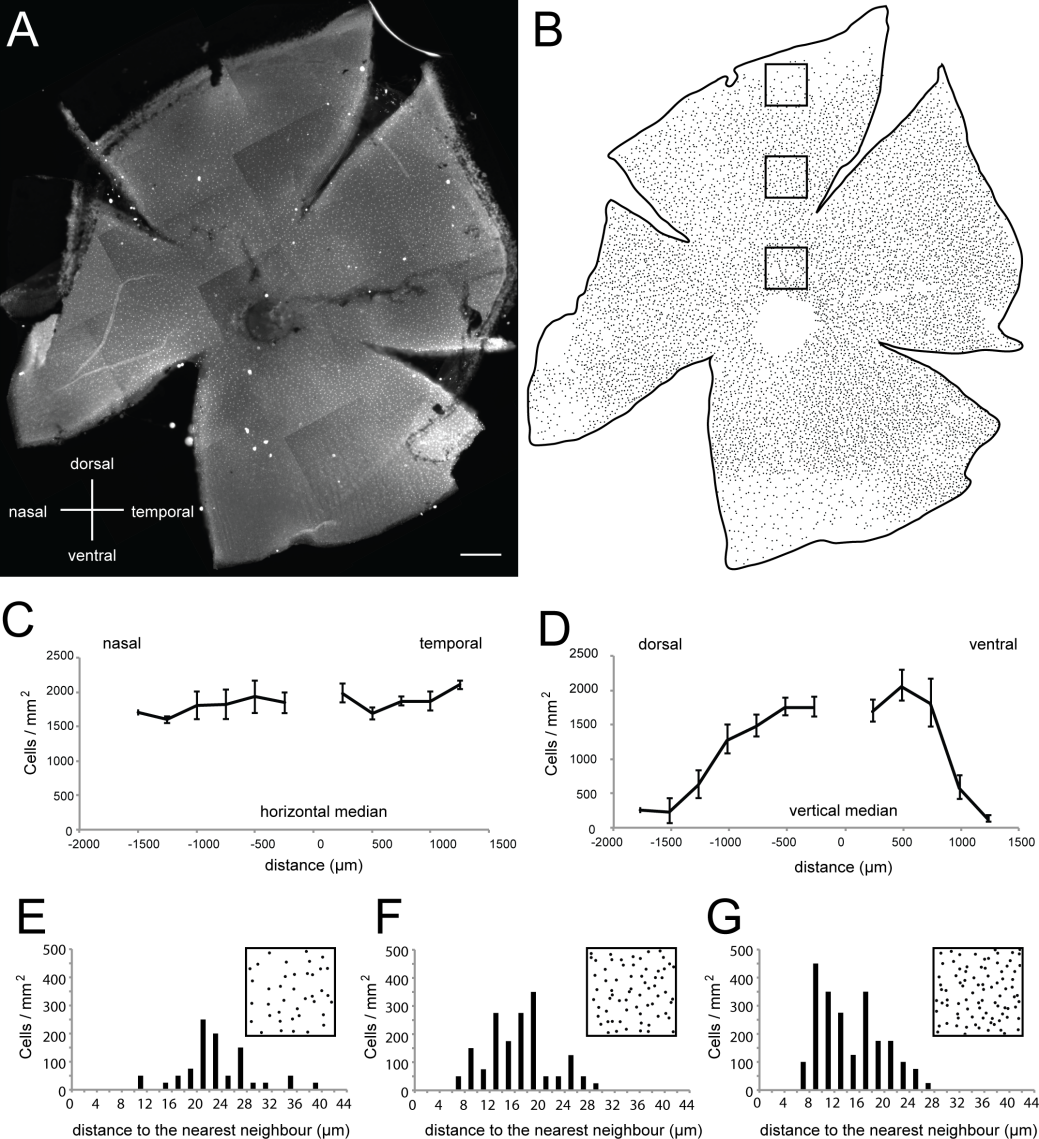
Secretagogin positive cells from a regular mosaic with highest density along the horizontal midline. (A, B) Micrograph collage and schematic showing secretagogin immunostaining in the inner nuclear layer of a flat mounted whole adult zebrafish retina. (C, D) Density of secretagogin labeled cells across the nasal-temporal (F) or dorsal-ventral (G) axes indicate high even density along the horizontal meridian, with the density along the dorsal-ventral axis peaking in central retina and decreasing towards the periphery (n = 20–21 ROIs for each of n = 3 adult eyes). Density was counted in ROIs (200 μm x 200 μm) every 250 μm until the edge of the retina. (E–G) Individual examples showing nearest neighbor analysis of region of interest (200 μm x 200 μm) indicated by boxed regions in B. Secretagogin labeled cells at any eccentricity are distributed regularly. ROIs were located at 250 μm, 750 μm and 1250 μm distance from the optic nerve center. Scale bar (A) is 200 μm.

The distribution and density of SCGN+ neurons was quantified in retinal whole mounts. The SCGN+ cells were present in all retinal regions (Fig 3A and 3B). The density of SCGN+ changed along the central–peripheral axis in the vertical, but not horizontal meridian (Fig 3C and 3D). Across the horizontal median from nasal to temporal retina, SCGN+ cell density was relatively stable (1838 ± 43 SEM, range 1600–2108 cells / mm^2^, Fig 3C). However, the density differed markedly along the vertical median (719 ± 207 SEM, range 125–2058 cells / mm^2^, Fig 3D), being lowest at the most peripheral regions. Across both meridians, the expected density gap in the center reflects the optic nerve head location. In summary, whilst widespread in distribution across all of the retinal quadrants, SCGN+ density is specifically reduced in the dorsal and ventral periphery.

The nearest neighbor analysis was utilized to characterize the regulatory of the SCGN+ cell mosaic across the different retinal locations using 200 μm square ROIs centered around 250 μm, 750 μm and 1250 μm distance from the optic nerve head center (Fig 3B and 3E–3G n = 3 ROIs from 3 adult zebrafish eyes). At this size, ROIs near the center of the retina contained ~80 cells, whereas the same sized ROI in the far peripheral regions contained between 9–35 cells, depending on the quadrant. Nearest neighbor analysis (as per Wässle and Riemann, [60])revealed that regardless of the observed differences in density across the central-peripheral gradient particularly in the vertical meridian, the regularity indices (RIs) for each region consistently showed a non-random distribution at > 1 (at 250 μm RI = 2.5, at 750 μm RI = 5, at 1250 μm RI = 3, Fig 2E–2G).

Within the retina, many neural subtypes show regular tiling, such that the visual circuits they are part of are able to sample visual information relatively equally regardless of the spatial location in the outside receptive field. The relatively regular distribution of SCGN+ cells suggests that SCGN+ cells comprise probably one, if not very few distinct amacrine subtypes.

### Co-localization of secretagogin and other calcium binding proteins

**Fig 4:**
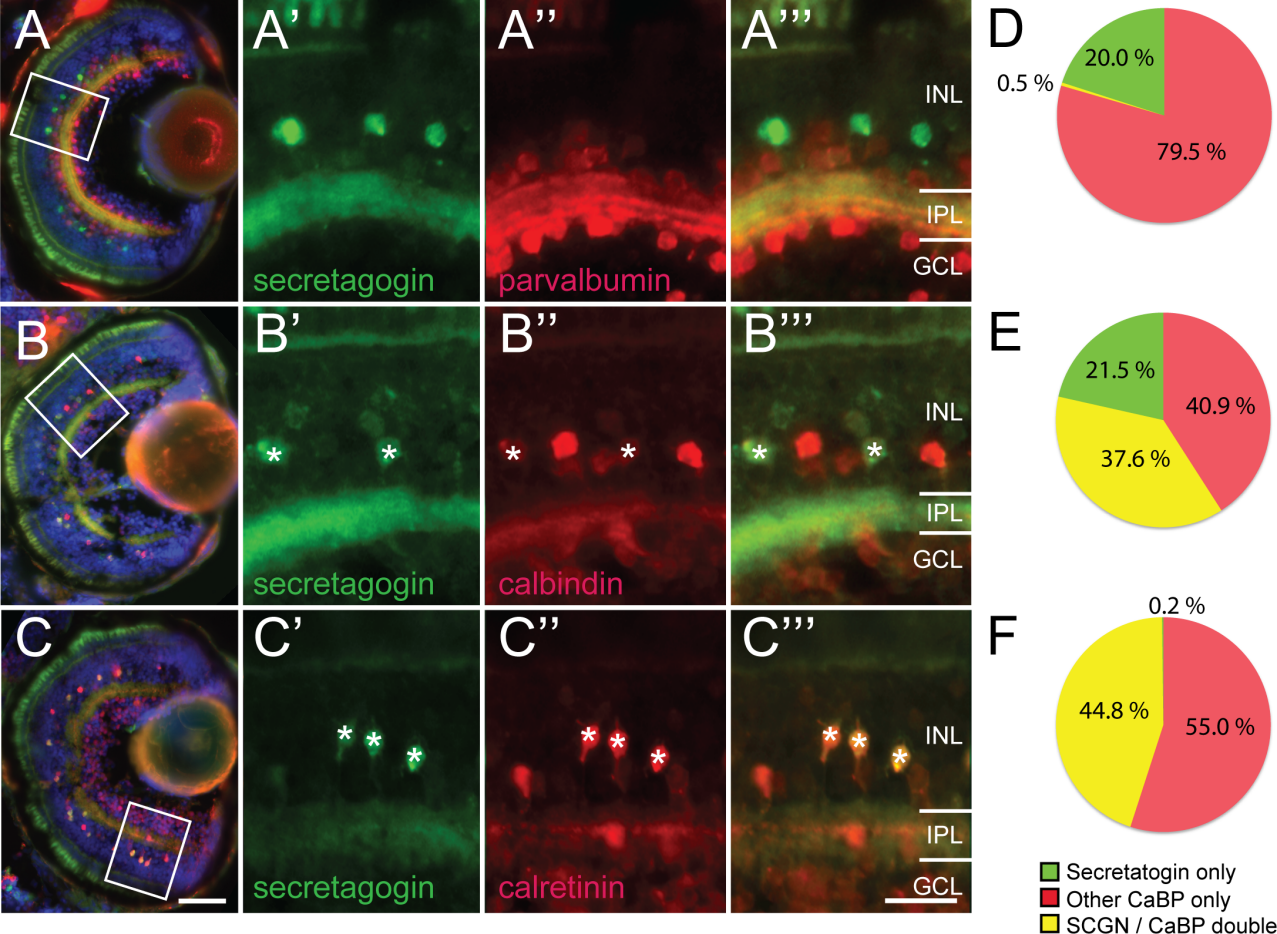
Comparative expression of secretagogin and other calcium binding proteins within the zebrafish retina. (A–C) Micrographs showing cross-sections through zebrafish retina at 5 days postfertilization. Higher magnification of boxed regions in each row show secretagogin expression in green and other calcium binding protein expression in red: Parvalbumin (PV–A), Calbindin (CB–B), Calretinin (CR–C). (D–F) Pie charts show quantification of singe and double labeling (asterisks). Secretagogin labeled cells are mutually exclusive from parvalbumin (D) expressing cells, but overlap partially with calbindin (E) and represent a subpopulation of calretinin (F) expressing cells. Scale bar (C) for A–C is 50 μm, scale bar (C”‘) for A’–C”‘ is 20 μm.

The co-labeling between secretagogin and well characterized calcium binding proteins parvalbumin (PV), calbindin (CB) and calretinin (CR) was quantified, to assess whether they are expressed by distinct subpopulations of amacrine cells. SCGN was only rarely co-expressed with parvalbumin (2% **±** 0.9% SEM of SCGN+ were PV+, n = 1463 cells from 7 larvae, Fig 4A) and overall SCGN cells labeled substantially less cells than PV (20.7% ± 1.3% SEM SCGN only vs. 78.8% ± 1.3% PV only). Thus, PV and SCGN label mutually exclusive amacrine cell populations. Unexpectedly, there was substantial overlap of SCGN expression with calbindin (Fig 4B) and calretinin (Fig 4C). The majority of SCGN+ cells also expressed calbindin (64% ± 3% SEM of SCGN+ expressed CB, n = 484 cells from 6 larvae). Of the total calbindin population, about half co-expressed SCGN (48% ± 3.1% SEM of CB+ expressed SCGN). Almost all of the SCGN+ expressed calretinin (99.8% ± 4.4% SEM of SCGN+ were CR+, n = 531 cells from 7 larvae). Additionally, the calretinin population was more than twice the size, with the SCGN+ co-labeled proportion making up 45.5% ± 2% SEM of the total CR+ population. Consistent with these results, parvalbumin labeled amacrine cell populations showed very little co-localization with either calbindin (5.4% ± 1% SEM, n = 783 cells from 16 larvae) or calretinin (8.7% ± 0.9% SEM, n = 754 cells from 12 larvae), as previously described [61]. Thus, unlike reported for other species and in other CNS regions, there is substantial overlap of SCGN+ expression in neuron types that also express calbindin and calretinin, but not parvalbumin.

### Co-localization of secretagogin and amacrine subtype markers

**Fig 5:**
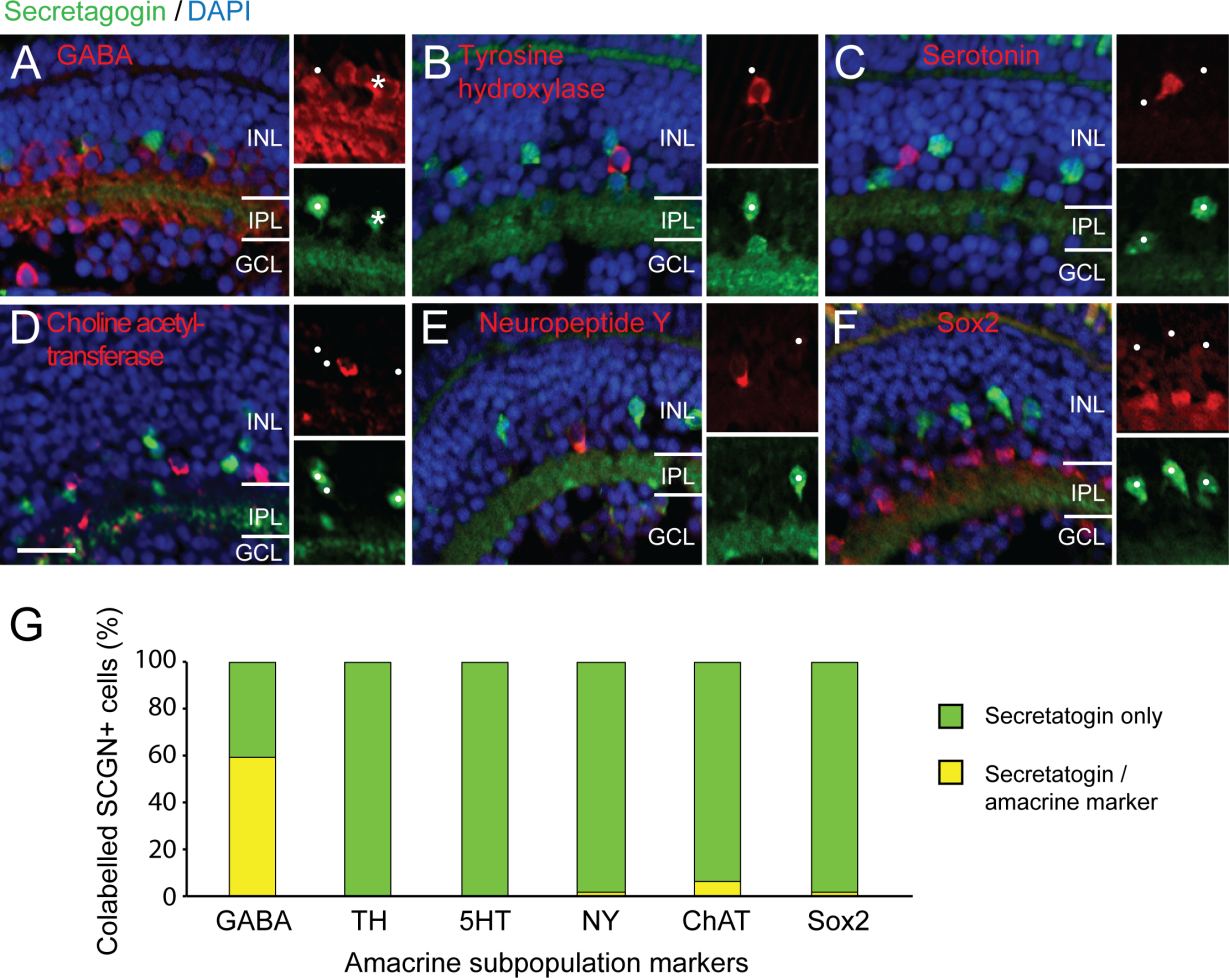
Secretagogin amacrine subtype markers and other calcium binding proteins within the zebrafish retina. (A–F) Micrographs showing cross-sections through zebrafish retina at 5 days postfertilization. Insets show separate red and green channels of subregion of the double channel images. Co-labeling (asterisks) of secretagogin—SCGN (green) with amacrine subtypes markers (red): GABA (A), tyrosine hydroxylase–TH (B), serotonin– 5HT (C), choline acetyltransferase–ChAT (D), neuropeptide Y—NY (E), Sox2 (F). Most SCGN+ cells show little co-localization with other markers (white dots). (G) Graph shows proportion of secretagogin cells that also co-label for the other amacrine markers (asterisks). Scale bar (D) for A–F is 20 μm.

In order to further characterize the possible subtypes of amacrine cells that specifically express SCGN, double label immunohistochemical quantification was carried out with other characterized amacrine subtype or subpopulation (possibly multiple subtype) markers in the zebrafish retina (Fig 5). Secratagogin showed very little to no co-localization with subtype markers including tyrosine hydroxylase, serotonin, neuropeptide Y, choline acetyltransferase or sox2 (range 0–6% co-labeling, n range = 146–489 SCGN+ cells from 16–50 larvae, Fig 5G). A total of 59.4% of SCGN+ cells did also show GABA immunoreactivity. The lack of substantial co-localization with other known subtype specific markers indicates that secretagogin can be used to identify a mutually exclusive amacrine population. Interestingly, the proportion co-localized with GABA, a major inhibitory neurotransmitter expressed in the majority of amacrine subtypes in the zebrafish retina [59], was neither 0% nor 100%. Because cells belonging to the same neural subtype generally express the same complement of genes including neurotransmitters, this result suggests that secretagogin may label a non-GABAergic and a GABAergic amacrine subtype population.

## Discussion

In this work, we provide the first characterization of SCGN protein in retinal neuron subtypes in the zebrafish, including the developmental onset of SCGN and a thorough quantification of distribution, density, stratification and subtype marker co-localisation with a particular focus on other known calcium binding proteins. In the zebrafish retina, SCGN labeled a novel subpopulation of amacrine interneurons, likely consisting of the two subtypes described with neurites stratifying in the centre of the inner plexiform layer. This provides the fundamental basis for comparative functional studies to start assessing the role of different calcium binding proteins in neurons.

### Developmental secretagogin expression

The first detectable SCGN immunoreactivity in retinal neurons occurred within the forming amacrine layer at 3 dpf, around the developmental stage at which visual responses can first be observed in zebrafish larvae [58]. The location of SCGN labeling at this time confirms expression in the amacrine cell layer, suggesting that SCGN plays little role in the early developmental stages of differentiation, which include cell cycle exist, and cellular migration of amacrine cells, complete by 60 hpf in this central retinal region [59]. The timing of SCGN does coincide with retinal calcium waves initiated by retinal bipolar cells between 2.5–3.5 dpf in zebrafish [62], which could contribute to refinement of visual circuits in higher visual centers, as described for other vertebrates (reviewed in [63, 64]). In mouse, SCGN expression is also described maturing neurons between P4 and P6 [30, 34] which is just prior synaptogenesis and coincides with calcium wave occurance [64]. Thus, regardless of cell type expression, the emergence of SCGN correlates well to a key developmental stage during retinogenesis within maturing neurons.

### Distribution, density and subtype identity of secretagogin labeled amacrine cells in zebrafish

The density of SCGN+ cells becomes established during the first 5 dpf. Wholemount characterization in adult zebrafish retina revealed a regular somal distribution of SCGN+ cells with regulatory indices ranging from 2.5–5 in the analysed regions of interest, which is consistent with labeling of a single subtype. Some regional density differences were observed, specifically with lowest densities in the dorsal and ventral periphery. Other retinal neuron subtype markers such as parvalbumin have previously been shown to display regional density differences, peaking in the temporal ventral quadrant [61] and a central to peripheral density reduction is consistent with retinal organisation across vertebrates. However, amacrine interneurons in vertebrates including zebrafish can generally (but not always, see [65]) be labeled with GABA or glycine inhibitory neurotransmitter markers ([59], reviewed in [66]). Whilst these neurotransmitters are expressed by multiple subtypes, a given subtype is generally expected to completely co-label with either GABA or glycine or neither. In mammals, narrow-field (small neurite arbor) amacrine subtypes tend to be glycinergic and wide-field amacrine subtypes tend to be GABAergic [67], though this does not seem to hold based on current data in zebrafish [59]. We observed that GABA co-labelled only with ~60% of SCGN+ amacrine cells. A similar proportion co-labeled with calbindin and almost all of these SCGN+ also co-expressed calretinin. This could either mean that a single SCGN+ amacrine subtype shows heterogeneity ina number of other markers (differ in protein expression including neurotransmitter), or that at least two SCGN+ amacrine subtypes are labeled. Using mosaic labeling techniques or intracellular injection approaches, the morphological features of individual cells could help distinguish these possibilities. In either case, the neurites of all SCGN+ amacrine cells only stratify in one distinct band in the center of the inner plexiform layer. Within this band, SCGN+ could receive input from a variety of different bipolar cells identified in zebrafish [68], including from the monostratified S4 bipolar cell types or from three potential multi-stratified bipolar cells (types S1/S4, S4/S6 or S2/S4/S6). While ganglion cells have been morphologically identified, in the zebrafish, their functional contributions to different visual modalities and mammalian homologues have not been characterized. Nonetheless, three diffuse or multi-stratified ganglion cell types (types 7, 8, 9) or two bistratified ganglion cell types (types 10 and 11) co-stratify with SCGN labeled amacrine cells [69]. Based on characterized amacrine cell types [59], SCGN+ is likely to label the narrow-field varicose 4 monostratified amacrine cell and possibly the medium or wide-field smooth monostratified subtypes that are found in the same plexiform sublayer. These do not co-label with other known subtype (rather than subpopulation, i.e. multiple subtypes) markers in the zebrafish retina and thus SCGN+ represents a novel label that complement current tools to identify a unique zebrafish amacrine subpopulation.

### Secretagogin and other calcium binding proteins

The first stages of visual processing in vertebrates are carried out by distinct subtypes of the main five retinal neuron types. The zebrafish contains at least 65 of these subtypes [59, 68–71], which is comparable to the variety found in other vertebrates [72]. However, while the functional role may be equivalent, the subtypes are not directly comparable across species. Thus, the identity of the secretagogin expressing retinal neurons we describe in zebrafish may not have a direct homologues in other vertebrates. Interestingly, the subtypes of neurons expressing secretagogin as well as other CaBP varies across retinas of different vertebrates, even across different mammalian species. Retinal secretagogin expression has been reported in bipolar cells in mice [30, 34], rats & rabbits [30], macaques [48] and ground squirrels [50], in photoreceptors in humans [49], and in various amacrine populations in marmosets [31], macaques [48] and humans [49], as we have found in the current study in zebrafish. Despite the wealth of data about the temporal and cell type specific expression of CaBP, still little is known about their function. The large variety of expression of the relatively newly identified secretagogin protein and other CaBPs observed in different cells across different species no single CaBP has a highly conserved function within any specific neural cell population.

In zebrafish retina, expression of various calcium binding proteins have been identified in photoreceptors, bipolar and amacrine cells [11]. Although CaBPs have different affinity for Ca^2+^, different roles (particularly as buffers versus sensors), and have been implicated in different functions, the large variability in their subtype expression pattern across species imply that they can either carry out similar roles or that they have non-essential possibly mainly modulatory roles. General Ca^2+^ dysfunction has been implicated in neurodegenerative disorders and can occur through dysregulation of any of the CaBPs [73, 74]. The specific function of described CaBPs in the vertebrate retina remains unknown (reviewed in [22]). With the genetic tools to perform reverse genetic experiments easily in the well characterized zebrafish retina model, loss of CaBP phenotypes can now be compared with the described wild type pattern characterized in this study to start dissecting out the role, redundancy and cross-regulation of these CaBPs. While secretagogin has been reported to generally show little co-expression with other CaBPs [25, 26, 33–35, 49] substantial co-expression has been reported for specific CNS regions [36, 37], as we have found in the current study between secretagogin and both calbindin or calretinin within the retinal amacrine cells. Interestingly, the pattern of co-expression of calcium binding proteins may also be correlated to differential electrophysiological responses [36], though whether this is a general pattern across CNS areas remains to be determined. Additionally, although both calbindin and calretinin antibodies were generated in rabbit, a substantial proportion of amacrine cells must co-express, these, given that most SCGN+ cells expressed calretinin and over half of these also co-expressed calbindin. These could carry out distinct or redundant roles within the cells. It will be interesting to contrast how loss of for instance secretagogin affects the development and function of neuronal subtypes that co-express other known CaBPs versus those that do not. Given the amenability of the zebrafish model for such studies, the SCGN characterization presented here will allows us to assess consequences of functional manipulations of CaBP.
